# Nanopore sequencing with T2T‐CHM13 for accurate detection and preventing the transmission of structural rearrangements in highly repetitive heterochromatin regions in human embryos

**DOI:** 10.1002/ctm2.1612

**Published:** 2024-03-06

**Authors:** Qiuping Xia, Taoli Ding, Tianli Chang, Jiangxing Ruan, Ji Yang, Menglin Ma, Jiaqi Liu, Zhen Liu, Shujing Jiao, Jian Wu, Jun Ren, Sijia Lu, Yanping Li, Zhongyuan Yao

**Affiliations:** ^1^ Reproductive Medicine Center Xiangya Hospital Central South University Changsha China; ^2^ Yikon Genomics Company, Ltd. Suzhou China

**Keywords:** heterochromatin, nanopore sequencing, structural rearrangements, T2T‐CHM13

## Abstract

**Background:**

Structural rearrangements in highly repetitive heterochromatin regions can result in miscarriage or foetal malformations; however, detecting and preventing the transmission of these rearrangements has been challenging. Recently, the completion of sequencing of the complete human genome (T2T‐CHM13) has made it possible to accurately characterise structural rearrangements in these regions. We developed a method based on T2T‐CHM13 and nanopore sequencing to detect and block structural rearrangements in highly repetitive heterochromatin sequences.

**Methods:**

T2T‐CHM13‐based “Mapping Allele with Resolved Carrier Status” was performed for couples who carry structural rearrangements in heterochromatin regions. Using nanopore sequencing and the T2T‐CHM13 reference genome, the precise breakpoints of inversions and translocations close to the centromere were detected and haplotypes were constructed using flanking single‐nucleotide polymorphisms (SNPs). Haplotype linkage analysis was then performed by comparing consistent parental SNPs with embryonic SNPs to determine whether the embryos carried hereditary inversions or balanced translocations. Based on copy number variation and haplotype linkage analysis, we transplanted normal embryos, which were further verified by an amniotic fluid test.

**Results:**

To validate this approach, we used nanopore sequencing of families with inversions and reciprocal translocations close to the centromere. Using the T2T‐CHM13 reference genome, we accurately detected inversions and translocations in centromeres, constructed haplotypes and prevented the transmission of structural rearrangements in the offspring.

**Conclusions:**

This study represents the first successful application of T2T‐CHM13 in human reproduction and provides a feasible protocol for detecting and preventing the transmission of structural rearrangements of heterochromatin in embryos.

## Graphical abstract

1. Nanopore sequencing with T2T‐CHM13 pioneers accurate blocking of heterochromatin rearrangements, reducing miscarriage or foetal malformations.
2. T2T‐CHM13‐based “Mapping Allele with Resolved Carrier Status” utilizes nanopore sequencing, precisely identifying breakpoints close to the centromere and constructing haplotypes to prevent hereditary inversions and translocations.
3. This endeavour represents the pioneering utilization of T2T‐CHM13 in the realm of reproduction.

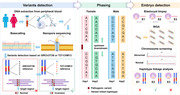

## 1 BACKGROUND

Chromosomal structural rearrangements, such as inversions and balanced translocations, are frequently observed in humans. They can cause fertility disorders and embryonic developmental abnormalities. Additionally, offspring carrying the same balanced translocation as their parents may also face pregnancy risks.^1^ Therefore, it is imperative to identify and prevent the inheritance of chromosomal structural rearrangements within families to ensure that future generations are not affected by this problem.

Preimplantation genetic testing for chromosomal structural rearrangements (PGT‐SR) has utilised various methods.^2^ Recent studies have shown that next‐generation sequencing (NGS)‐based methods, including “Micro‐Seq^3^” and “Mapping Allele with Resolved Carrier Status” (MaReCs),^4^ can differentiate between normal and carrier embryos through whole genome amplification using multiple biopsied cells.^5^, ^6^ However, these methods have certain limitations. For instance, they require the complete genetic information of the pedigree and may be difficult to apply to complex genomic regions owing to the short read lengths of NGS technology.^7^

In contrast, nanopore sequencing, a third‐generation sequencing (TGS) technology, offers long read lengths and lacks GC bias, making it ideal for spanning complex regions of the genome, such as those with high GC ratios and pseudogenes. It also offers unparalleled advantages in detecting structural rearrangements.^8^ Among the various techniques for detecting embryo structural rearrangements, MaReCs based on nanopore sequencing can comprehensively and accurately identify breakpoints (with single‐base accuracy) and directly construct haplotypes around the breakpoints. This eliminates the need for a proband or complete genetic information of the pedigree as a reference. By solely analysing samples from both males and females, we can determine whether the embryo carries paternal or maternal mutations. The implementation of MaReCs based on nanopore sequencing holds promise in simplifying PGT and benefiting a wider range of families. Nevertheless, detecting structural rearrangements in highly conserved repetitive heterochromatin such as telomeres and centromeres remains challenging.

The Telomere‐to‐Telomere (T2T) Consortium unveiled the 3.055 billion base pair sequence of the human genome, known as T2T‐CHM13, in March 2022, marking a significant milestone in achieving a truly complete human genome assembly.^9^ This genome provides gapless assemblies for chromosomes, corrected errors in the prior GRCh38 reference genome and added nearly 200 million base pairs of sequences, including in heterochromatic regions. T2T‐CHM13 is expected to reveal new information and mechanisms related to previously incomplete genomic regions and promote medical progress. Although this breakthrough is exciting, more attention needs to be paid to how TGS and T2T‐CHM13 can be specifically applied in clinical medicine. TGS and T2T‐CHM13 offer potential solutions for identifying and blocking the propagation of pathogenic mutations in heterochromatin for PGT‐SR.

In this study, we successfully demonstrated the effectiveness of utilising T2T‐CHM13‐based MaReCs to enable couples to conceive healthy babies without the pathogenic structural rearrangements inherited from their parents. Nanopore sequencing coupled with T2T‐CHM13 enabled precise identification of the breakpoints of inversions and translocations close to the centromere. Immediate phasing with flanking single‐nucleotide polymorphisms (SNPs) around the breakpoints was achieved and haplotype linkage analysis was performed by comparing consistent parental SNPs with embryonic SNPs to determine whether the embryos carried hereditary inversions or balanced translocations. Our findings represent a proof‐of‐concept for the practical applications of T2T‐CHM13 and TGS in human reproduction and provide technical guidance for the detection and prevention of structural rearrangements in heterochromatin regions in medical studies and clinical cases.

## 2 METHODS

### 2.1 Karyotype analysis

Peripheral blood was routinely cultured as cell fluid prior to cytological processing. The cell fluid was then pipetted into a centrifuge tube and centrifuged at 3000 rpm for 10 min. The supernatant was removed, and a 1:1 mixture of 0.4% potassium chloride and 0.4% sodium citrate, prewarmed at 37°C, was added to 8 mL supernatant and placed in a 37°C water bath (or oven) for 15 min. Subsequently, 1 mL of 3:1 methanol: glacial acetic acid fixative was added to the hypotonic cell fluid and after gentle mixing, the samples were centrifuged at 3000 rpm for 10 min. After removing the supernatant, 8 mL of fixative was added, and the solution was changed 30 min later. At least two fixative changes were required, followed by centrifugation at 3000 rpm for 10 min. After overnight incubation, the solution was centrifuged, and the upper layer of the fixative was aspirated. Eight to ten drops of a new fixative were then added, and the cell pellet was gently scattered into a cell suspension using a pipette. One to two drops were added to a slide that had been pre‐cooled. The slides were immediately flame‐dried using an alcohol lamp without allowing them to burn. The slide was baked in an oven at approximately 70°C for 2–3 h and left to cool naturally. It was then developed using 0.025% trypsin solution (pH 7.4+) made using normal saline, which was prewarmed at 37°C for 1–3 min. Finally, the cells were stained with Giemsa for 15 min.

### 2.2 Fluorescence in situ hybridization characterization and validation

Peripheral blood samples (0.3 mL) anticoagulated with heparin were collected and inoculated into 5 mL of RPMI‐1640 medium supplemented with PHA. The mixture was incubated for 69 h, followed by the addition of colchicine. After cytological treatment, a cell suspension (approximately 1×10^5^ cells/mL) was prepared. Subsequently, 5–6 µL of the suspension was dropped onto the centre of a slide, and dehydration was carried out using 70%, 90% and 100% ethanol for 3 min each. The slides could then be immediately subjected to hybridization or stored at −20°C until further use.

For hybridization, the slides were removed from the freezer, dehydrated in 100% ethanol and air‐dried. Subsequently, 80 µL of RNase A was added, and the slides were covered with wax film. Digestion was performed at 37°C for 1 h, followed by rinsing in phosphate‐buffered saline and subsequent dehydration in 70%, 90% and 100% ethanol for 3 min each. The slides were air‐dried for future use. The probes were denatured at 76°C for 5 min, immediately placed on ice for 5–10 min, and then incubated in a 37°C water bath for 30 min. Afterwards, the slides were denatured in 70% formamide or 2×SSC at 70°C for 2 min. The slides were then immediately placed in −20°C 70%, 90% and 100% ethanol for dehydration (3 min each) and air‐dried for future use. Subsequently, a denatured probe (6–7 µL) was added to the chromosomal specimen on the dried slide, and hybridization was performed for 16–18 h at 37°C in a wet box after blocking. The slides were washed twice in 50% formamide with 2×SSC for 5 min each at 42–45°C. Subsequently, they were rinsed twice in 0.1×SSC for 5 min each and dehydrated in 70%, 90% and 100% ethanol for 3 min each. Finally, the slides were air‐dried at room temperature and stained with DAPI for 15 min.

### 2.3 Intracytoplasmic sperm injection and biopsy

Obtained oocyte corona cumulus complexes were incubated for 2–4 h in an incubator and then transferred to prewarmed hyaluronidase dishes. Granulosa cells located in the cumulus and corona were gently removed by pipetting in Papanicolaou tubes for no more than 30 s. After retrieval, the oocytes were transferred to a fresh degranulation cell dish and washed several times. Subsequently, the granulosa cells surrounding the oocytes were carefully removed using a Papanicolaou tube of appropriate diameter by meticulous pipetting. MII stage oocytes were selected using an inverted microscope, transferred to an incubation dish and incubated for 2 h in an incubator. During intracytoplasmic sperm injection (ICSI), the first polar body of the oocyte was secured near the 12 o'clock position by using a holding needle. Sperm was carefully injected into the oocyte cytoplasm while ensuring that the spindle was not damaged. Following ICSI, oocytes were incubated to continue the culture process. Once the zygotes had developed into blastocysts on day 5 or 6, we utilised a biopsy needle to extract 3–5 trophectoderm (TE) cells from the blastocysts for MaReCs detection. The embryos were cryopreserved by vitrification before being placed in liquid nitrogen.

### 2.4 DNA extraction

Peripheral blood was collected and high‐molecular‐weight genomic DNA was extracted using the SDS method. The extracted DNA was further purified using a QIAGEN Genomic Kit (Cat#13343, QIAGEN) as per the manufacturer's standard operating procedure. A 1% agarose gel was used to assess the degradation and contamination of the extracted DNA. Subsequently, DNA purity was determined using a NanoDrop One UV‐Vis spectrophotometer (Thermo Fisher Scientific) with OD 260/280 values ranging from 1.8 to 2.0 and OD 260/230 values between 2.0 and 2.2. Further, DNA concentration was measured using a Qubit 3.0 Fluorometer (Invitrogen).

### 2.5 Library preparation and nanopore sequencing

For the Oxford Nanopore Technology library preparations, each sample was provided with 2 µg of DNA as input material. The BluePippin system (Sage Science) was used for the qualified size selection of long DNA fragments. Next, the ends of the DNA fragments were repaired and subjected to ligation using the NEBNext Ultra II End Repair/dA‐tailing Kit (Cat# E7546; NEB). The adapter in the LSK109 Kit was used for further ligation reactions, and the size of the resulting library fragments was quantified using a Qubit 3.0 Fluorometer. Subsequently, sequencing was performed using a PromethION sequencer (Oxford Nanopore Technologies).

### 2.6 SNP genotyping

To conduct haplotyping analysis, SNPs were identified in each sample using the Infinium Asian Screening Array (Cat# 20016317; Illumina, Inc.), a high‐throughput genotyping array, following the manufacturer's instructions.

### 2.7 Mapping, detection of structural variants, phasing and haplotype linkage analysis

Raw reads in FASTQ format were obtained from the electrical signal produced by PromethION using Guppy basecalling software (v5.0.16). To ensure the quality and reliability of the analysis, NanoFilt (v2.8.0) (https://doi.org/10.1093/bioinformatics/bty149, Flanders Institute for Biotechnology) was used to remove low‐quality (Qphred < = 7) and short reads (< 1000 bp) from the raw data. Additionally, 50 bp bases in the head/tail were trimmed.

We used Minimap2 to align the reads with reference genomes, including GRCh37, GRCh38 and T2T‐CHM13. The parameter used for Minimap2 was ‐ax map‐ont ‐L –MD ‐Y ‐t 20. Subsequently, we transformed the SAM format into BAM using SAMtools (v1.2) (https://doi.org/10.1093/gigascience/giab008).

We processed BAM files through Sniffles (https://doi.org/10.1038/s41592‐018‐0001‐7, Human Genome Sequencing Center, Baylor College of Medicine), with the parameter of ‐t 12 –min_support 4 –num_reads_report −1. The preliminary structural variant (SV) results underwent initial screening based on high‐quality variant reads (DV) and reference reads (DR). Additional screening criteria were added to improve the specificity of the detection. 1) Mutations with a DV number greater than 4 were retained; 2) SVs that were supported by a proportion of reads (DV/(DV+DR)) exceeding 10% were retained; 3) In particular, mutations longer than 5 Mb would only be retained if detected by karyotype analysis. These measures, along with the karyotype diagnosis report, ensure a higher confidence in the reported variants. PEPPER‐WhatsHap‐DeepVariant (r0.7‐GPU) (https://doi.org/10.1038/s41592‐021‐01299‐w, UCSC genomics institute and the Genomics team in Google Health) is a tool that can be used to report single‐base mutations and indels in samples, while simultaneously obtaining haplotype results. Using the BAM file as the input with the following parameters: ont_ r9_ guppy5_ sup ‐g –phased_ Output—t 12, a VCF file that includes phasing information can be generated. Classification results for the target region were obtained using Python.

Before the annotation of SNPs and SVs, we converted the genomic coordinates to the GRCh37 human genome version utilizing the UCSC liftOver tool (UCSC Genome Browser, https://genome.ucsc.edu/). For the functional annotation of SNPs, we utilized ANNOVAR, a software tool designed to annotate genetic variants detected from diverse genomes (https://doi.org/10.1093/nar/gkq603). For DELs, DUPs, INSs, INVs and BNDs in the SV list, we performed AnnotSV (https://www.lbgi.fr/AnnotSV/) for the annotation. For DELs and DUPs, a risk rating (ACMG class) was provided to characterize the impact of target SVs (https://doi.org/10.1038/s41436‐019‐0686‐8).

For reliable variant results, we plotted the altered chromosomes based on the T2T‐CHM13 Cytoband provided by the UCSC Genome Browser. Notably, only mutations exceeding 100 kb are displayed in the figures.

We used a likelihood‐based haplotyping approach with a hidden Markov model strategy to identify the most likely haplotype configuration of the embryos and other non‐probands.

## 3 RESULTS

### 3.1 Description of patients and karyotypic findings

**FIGURE 1:**
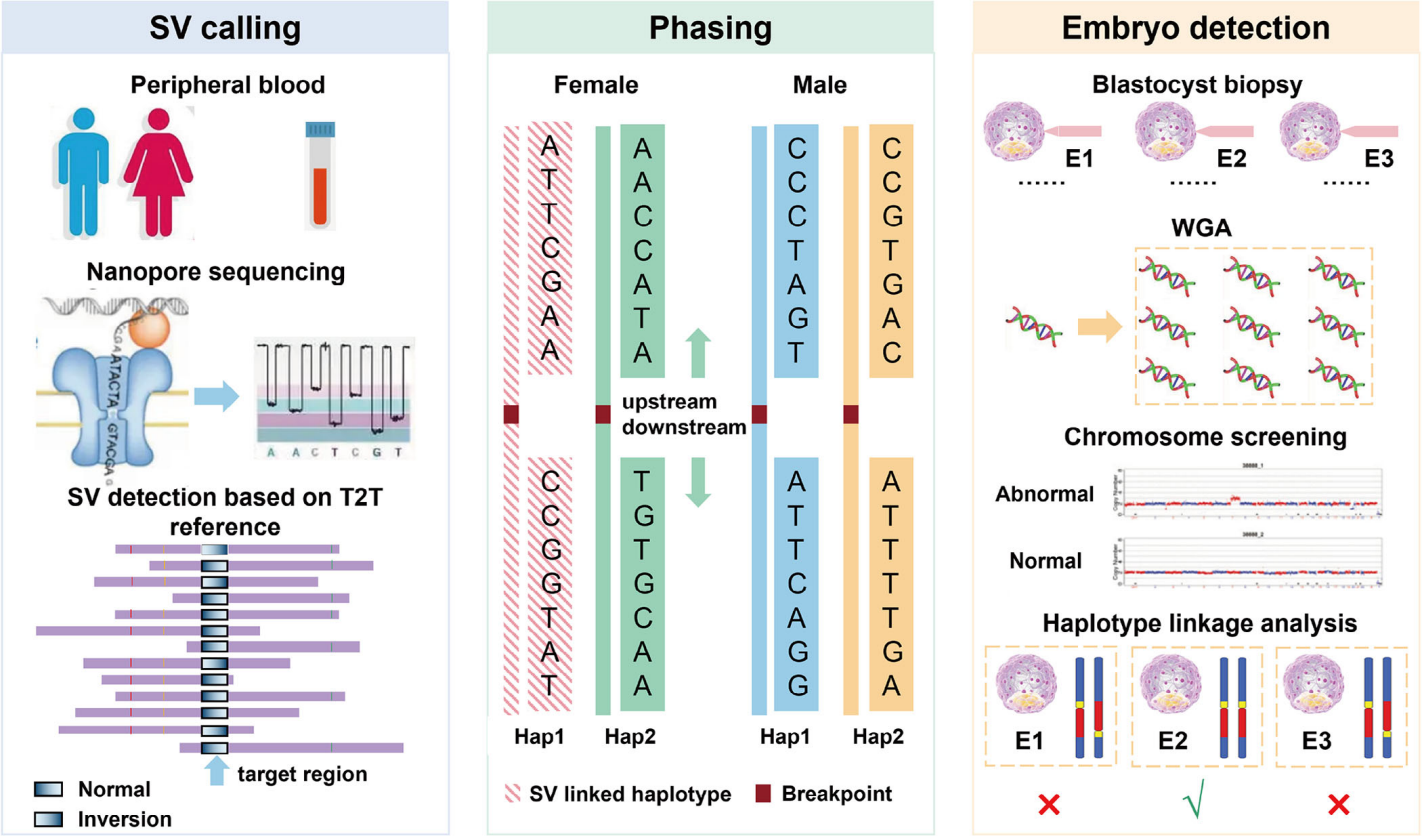
Schematic of mapping allele with resolved carrier status (MaReCs) flow based on third‐generation sequencing (TGS) and the Telomere‐to‐Telomere (T2T) CHM13 reference genome. SV, structural variant; WGA, whole genome amplification.

To investigate the detection and prevention capabilities of nanopore sequencing and T2T‐CHM13 for structural rearrangements close to the centromere, MaReCs were conducted on two families with inversion and reciprocal translocation carriers, respectively. The technical principles of nanopore sequencing and the pathways of MaReCs combined with nanopore sequencing and T2T‐CHM13 are shown in Figure 1.

**FIGURE 2:**
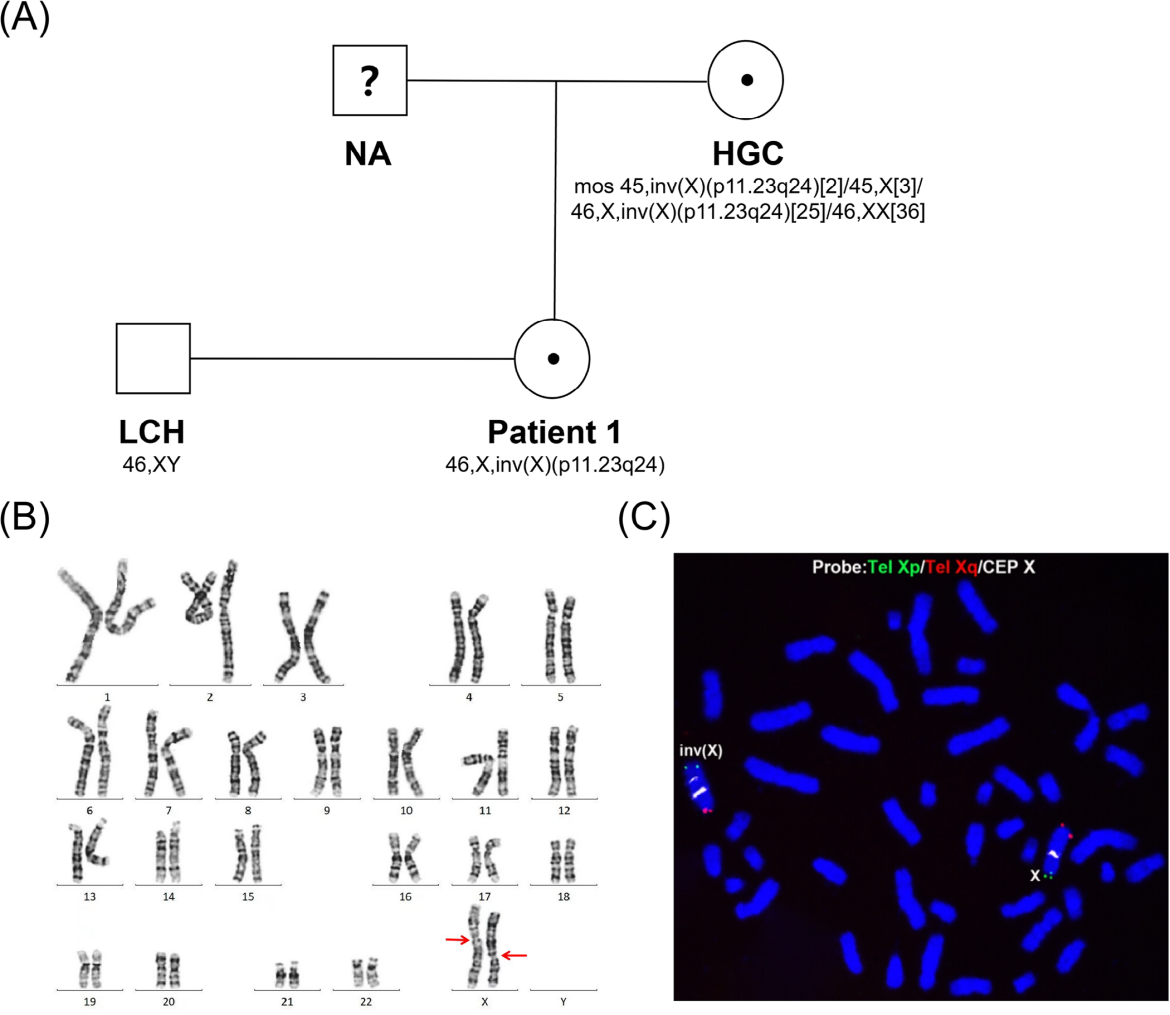
Genetic pedigree mapping and karyotype results of pedigree 1. (A) Genetic pedigree mapping of patient 1. The father of patient 1 had passed away. Patient 1's mother (HGC) revealed a complex karyotype of mosaic mutations. Both patient 1 and her mother carried an X‐linked inversion. Patient 1's husband (LCH) had a normal karyotype. (B) Karyotype result of patient 1 based on chromosome G‐banding. (C) Fluorescence in situ hybridisation (FISH) results were obtained from patient 1's peripheral blood cells using the Tel Xp (green)/Tel Xq (red)/CEP X (white) probe set.

In the first pedigree, patient 1 was 34 years old and her husband was 28 years old. Following a missed abortion in 2015, we conducted a series of investigations to determine its cause. Subsequent examinations revealed that patient 1 was an inversion carrier with a high‐resolution karyotype of 46,X,inv(X)(p11.23q24), which was identified as a possible cause of the miscarriage. However, her husband had a normal 46,XY karyotype. In addition, her father had passed away and karyotype analysis of her mother revealed a complex mosaic result. The genealogical tree and karyotype results for the first pedigree are shown in Figure 2A,B.

To confirm the accuracy of the karyotyping results, we conducted fluorescence in situ hybridisation (FISH) using the Tel Xp/Tel Xq/CEP X probe set on peripheral blood cells from patient 1. In Figure 2C, we observed 30 mitotic phases and detected 2 white signals of the CEP X probe on the inverted X chromosome. In contrast, the normal X chromosome displayed only one white signal. The FISH results (Figure 2C) confirmed that patient 1 was indeed an inversion carrier. The karyotype of patient 1, as determined by FISH, was 46,X,inv(X)(p11q22).ish inv(X)(Tel Xp+,Tel Xq+,CEP X++), which differed slightly from the results obtained by chromosome G‐banding.

Patient 2 in the second pedigree was a 30‐year‐old male, who had a balanced translocation with a karyotype of 46,XY,t(13;17)(q11;q11.2). The female in the pedigree was 28 years old and had a normal 46,XX karyotype. However, karyotype results for the parents of patient 2 were unavailable.

### 3.2 TGS‐ and T2T‐CHM13‐based detection of structural rearrangements

Libraries were constructed using nucleic acids extracted from the peripheral blood of patients 1 and 2. Sequencing was performed using the PromethION 48 Kit (Oxford Nanopore Technologies). After data filtering, the mean read length for patient 1 was 18 609 bp and the N50 was 22 476 bp, with an average sequencing depth of 27.87× for the whole genome. Patient 2 had a mean read length of 18 021 bp and an N50 of 23 099 bp, with an average sequencing depth of 34.07× for the whole genome.

First, GRCh37 and GRCh38 were used as reference genomes to detect pathogenic mutations in patient 1 that were diagnosed by karyotyping. However, no credible inversions were detected in the target regions of p11.23 and q24 on the X chromosome using Sniffles software.^10^ This may be attributed to unknown gaps in the sequence of centromeres on the X chromosome in GRCh37 and GRCh38.^9^ Sequencing reads cannot be aligned to the centromere, which hinders the detection of related structural rearrangements.

**FIGURE 3:**
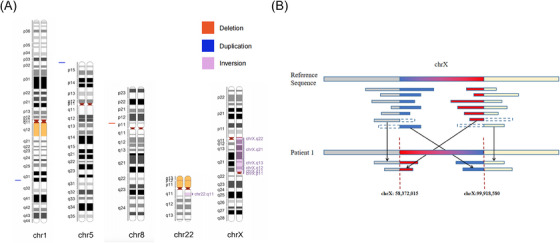
Display of mutation detection results for patient 1. (A) The major filtered mutations exceeding 100 kb. (B) Schematic diagram of target mutation, showing the inversion in chromosome (chr) X.

Next, the latest version of the T2T‐CHM13 v2.0 genome was used as a reference to perform sequence alignment and mutation detection. First, we conducted a comprehensive analysis to detect various mutations across the whole genome based on the T2T‐CHM13 reference genome and nanopore sequencing data. The statistical results of these mutations are presented in Table S1. Second, specific criteria outlined in the “METHODS” section were applied to filter the detected mutations. Notably, mutations exceeding 5 Mb were retained only if they were identified through karyotype analysis. The major filtered mutations exceeding 100 kb are presented in Figure 3A. Third, we annotated the filtered mutations to obtain pertinent information such as risk classification (ACMG class), associated genes and overlapping regulatory elements. The major filtered mutation annotation results for patients 1 and 2 are shown in Table S2. Detailed annotation results for all filtered mutations are provided in Tables S3–S6.

With the assistance of the Integrative Genomics Viewer (IGV) diagram, we successfully called the heterozygous pathogenic inversion with single‐base resolution, consistent with the results obtained from karyotype analysis. One breakpoint of the inversion was identified at 58 372 015 on the centromere (Xp11.1). The other breakpoint was located at 99 918 578 on the long arm of the chromosome (Xq22.1), causing disruption in *ARMCX4*. The original breakpoint position in the T2T‐CHM13 reference (99 918 578) was converted to a position in the GRCh37 reference (100 729 404). The total length of the inversion was 41, 546, 563 bp. To provide a comprehensive presentation of the mutation, we generated a schematic diagram (Figure 3B). Additionally, we included the IGV plots for patient 1 (Figure S1) and provided information on unaligned bases.

To verify the feasibility of T2T‐CHM13 for balanced translocation, we performed a retrospective analysis using patient 2 as the subject.^11^ We had already identified the precise breakpoints of 26 208 295 (13q11) and 33 942 281 (17q11.2) using GRCh37. However, TGS revealed that the actual location of the breakpoint was some distance from the centromere, which was not entirely in line with the karyotype. Mutation detection was also performed using the T2T‐CHM13 reference, and the identified breakpoints were located at 24 844 058 (13q11) and 36 563 186 (17q11.2). The positions of the specific breakpoints varied using different reference genomes. In Figure S2A, we presented a schematic diagram illustrating the balanced translocation in patient 2 to provide a visual representation of the breakpoints on chromosomes 13 and 17. Additionally, we presented the IGV plots for patient 2 (Figure S2B,C), which offer a detailed view of the mismatch patterns near the translocation breakpoints.

### 3.3 Haplotype construction using SNPs sequenced by TGS

First, we performed phasing using breakpoints and flanking SNPs in the T2T‐CHM13 reference. Considering the embryonic SNPs obtained using ChIP (chromatin immunoprecipitation) under the GRCh37 reference genome, the parental haplotypes were transformed into the GRCh37 reference for subsequent haplotype linkage analysis. SNPs located within a 2 Mbp range upstream and downstream of the breakpoint on the same read or contig were classified as belonging to the same haplotype, typically hap1. SNPs that were not linked to the breakpoint were grouped into another haplotype, usually hap2.^12^, ^13^

We obtained 633 reliable SNPs within the 2 Mbp range to perform phasing for the breakpoint of 99 918 578 (patient 1) located on the long arm of the chromosome. The pathogenic haplotype was identified and labelled hap1. Some of the phased SNPs are shown in Table S7. Additionally, we identified 612 reliable SNPs for phasing based on TGS and T2T‐CHM13 for another breakpoint (58 372 015) of the inversion located on the centromere. However, the quality of the phasing was not sufficiently high because of the influence of the centromere. Thus, the haplotype of the centromeric region was not used in the subsequent haplotype linkage analysis of embryos.

Haplotypes were constructed using distinct flanking SNPs in the T2T‐CHM13 reference for the two breakpoints (24 844 058 and 36 563 186) of the balanced translocation of patient 2. Subsequently, the phased haplotypes were transformed into the GRCh37 reference to facilitate embryonic haplotype linkage analysis. The transformed haplotypes were consistent with those obtained using GRCh37 (Tables S8 and suppinfo12).

### 3.4 Embryonic haplotype linkage analysis and prenatal diagnosis

Blastocysts obtained via ICSI were biopsied to obtain TE cells, which were subsequently used for MaReCs. The first stage involved examining the embryos for chromosomal copy numbers, whereas the second stage involved using blastocysts with normal chromosome copy numbers to determine whether the embryos carried parental structural rearrangements through haplotype linkage analysis.

**FIGURE 4:**
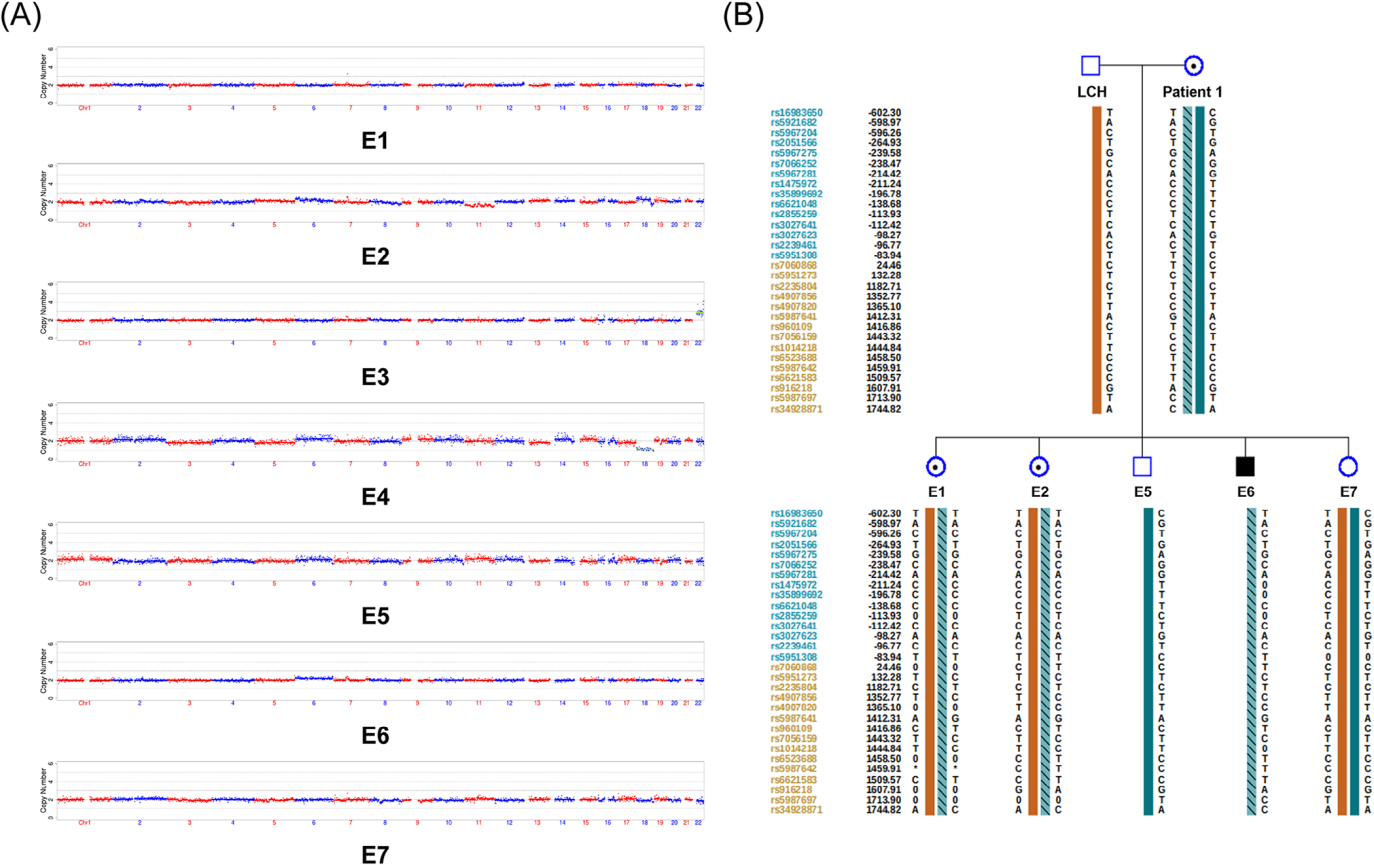
Haplotype linkage analysis related to the breakpoint of 100,729,404 (GRCh37) based on ASA single‐nucleotide polymorphisms (SNPs). (A) Copy number variation (CNV) results of embryos from patient 1. E1, E2, E5, E6, and E7 exhibit a normal CNV of 46,XN. E3 shows a result of 47,XN,+22(×3), and E4 shows a result of 45,XN,−18(×1). (B) Each coloured ribbon in the graph represents a haplotype. The shaded ribbon represents the pathogenic haplotype linked to the inversion, which is labelled as hap1 by default. SNP positions are annotated to the left of the graph. Positions marked in blue indicate a location upstream of the breakpoint, and those marked in yellow indicate a location downstream of the breakpoint.

In the first pedigree, 12 blastocysts were obtained from patient 1, seven of which were biopsied for MaReCs. Copy number variation (CNV) analysis revealed that five embryos (E1, E2, E5, E6 and E7) were normal. However, E3 showed a result of 47,XN,+22(×3) and E4 showed a result of 45,XN,−18(×1). The CNV results are shown in Figure 4A.

**FIGURE 5:**
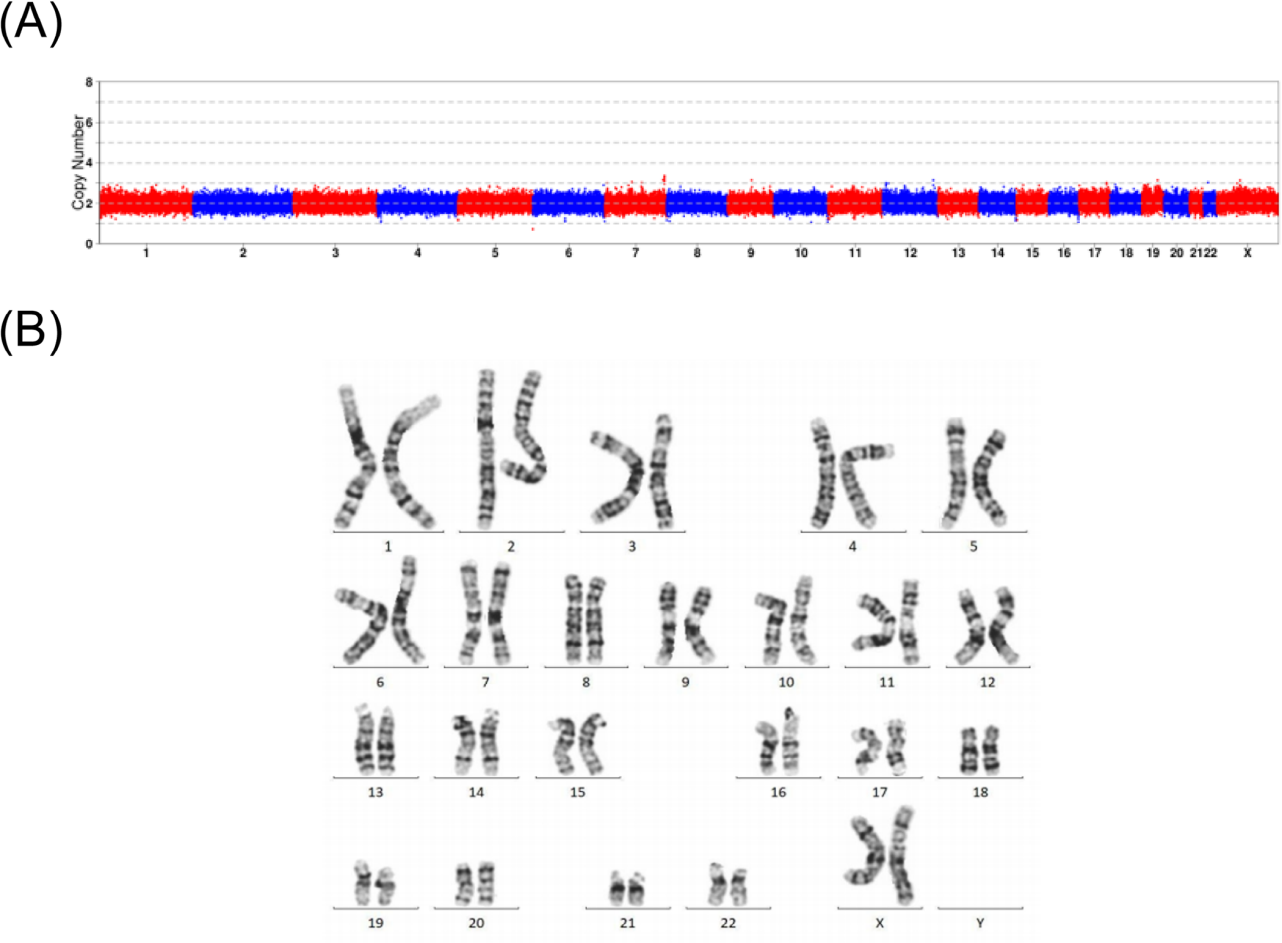
Prenatal diagnosis results using an amniotic fluid test in pedigree 1. (A) Copy number variation (CNV) result of embryo E7 from patient 1. (B) Result of karyotype analysis of embryo E7 from patient 1.

Next, we proceeded to the second stage using five blastocysts with normal chromosomal copy numbers. Furthermore, we obtained SNP information from other family members and embryos using an Infinium Asian Screening Array (ASA, Illumina) chip, in addition to patient 1. After filtering out low‐quality SNPs, we identified 32 reliable SNPs located within the 2 Mbp range of the inversion breakpoint at 99 918 578.^14^, ^15^ Haplotype linkage analysis was performed by comparing the coincident phasing SNPs of patient 1 obtained from TGS with those of embryos obtained from the ASA chip. Based on these 32 SNPs, we demonstrated that E1, E2 and E6 inherited the maternal inversion, whereas E5 and E7 did not. The results of haplotype linkage analysis based on SNPs are shown in Figure 4B. The breakpoint located at 58,372,015 around the centromere could not be used for the haplotype linkage analysis of embryonic SNPs. This was because the number of SNPs upstream of the breakpoint was insufficient owing to the influence of the centromere. E7 was successfully transferred to patient 1′s uterus with complete informed consent, resulting in a singleton pregnancy. Subsequent prenatal diagnosis using amniotic fluid confirmed a normal CNV and karyotype, and the offspring did not inherit the maternal inversion. The CNV and karyotyping results for the foetus are shown in Figure 5A,B.

In the second pedigree, 12 blastocysts from patient 2 and his wife were analysed. CNV analysis revealed that five embryos (A, B, C, K and L) exhibited normal ploidy. In the second stage, haplotype linkage analysis was performed using two‐phase haplotypes based on distinct breakpoints (24 844 058 and 36 563 186 in T2T‐CHM13). Consistent conclusions were obtained using different breakpoints, indicating that embryos B, C and K inherited paternal translocations, whereas embryos A and L did not. The details are shown in Tables S8 and 9. We also compared our findings with the previous results of haplotype linkage analysis directly under GRCh37 and found them to be consistent.

## 4 DISCUSSION

In clinical settings, patients commonly encounter miscarriages and foetal malformations resulting from chromosomal structural rearrangements such as inversion and translocation. Typically, these patients seek assistance through genetic counselling and NGS‐based MaReCs to ensure a healthy birth.^4^ However, conventional NGS and TGS‐based approaches are not effective in cases where translocation or inversion occurs in the centromere or telomere due to the unknown gaps in the GRCh37 and GRCh38 reference genomes, which hinder sequence alignment. MaReCs technology based on TGS can provide a solution for patients with de novo mutations or incomplete pedigrees.^16^, ^17^, ^18^, ^19^, ^20^, ^21^, ^22^

In our study, we innovatively used the latest T2T‐CHM13 v2.0 as a reference to successfully detect the precise breakpoints of the inversion and translocation occurring in the centromere using nanopore sequencing. We then constructed haplotypes to perform haplotype linkage analysis by comparing parental and embryonic SNPs. Finally, we transplanted the identified normal embryos, which were validated by prenatal diagnosis. This novel methodology allows physicians to accurately detect structural rearrangements in highly repetitive regions and identify whether embryos carry structural rearrangements. Our study provides a feasible protocol for medical and clinical PGT‐SR related to heterochromatin that cannot be performed using GRCh37 and GRCh38. To the best of our knowledge, this is the first report on the application of the T2T‐CHM13 reference in human reproduction.

In clinical applications, it is important to consider several technical aspects of this method. First, it was necessary to convert the haplotypes in the T2T‐CHM13 reference to those in the GRCh37 reference for subsequent embryonic haplotype linkage analysis after detecting the target mutation using the T2T‐CHM13 reference genome. However, if the target mutation or SNPs cannot be detected using GRCh37, this transformation is not possible because of the unknown gaps in GRCh37. Second, because of the difficulty in capturing SNP information in highly repetitive regions, there is a high probability that embryonic SNPs in the centromere or telomere will be insufficient when obtained through NGS or chips using GRCh37.

To address these challenges, our approach was to construct a haplotype for the inversion breakpoint located in the long arm of the chromosome. We detected the inversion breakpoint in the centromere and constructed haplotypes using TGS and the T2T‐CHM13 reference genome without performing embryonic haplotype linkage analysis. Therefore, our method is suitable for specific chromosomal structural rearrangements where only one end of the translocation or inversion is located in the heterochromatin region. With the ongoing development of TGS and T2T‐CHM13, MaReCs for Robertsonian translocation, circular chromosomes, and other complex structural rearrangements whose breakpoints are located in heterochromatin regions can be realised in the future. However, it is important to conduct further studies in larger cohorts to validate the clinical application of this approach.

## 5 CONCLUSIONS

In this study, we used MaReCs for couples with structural rearrangements in the heterochromatin regions. We successfully detected breakpoints and constructed haplotypes using flanking SNPs with the assistance of nanopore sequencing and the T2T‐CHM13 reference genome. We performed haplotype linkage analysis with embryonic SNPs to determine whether the embryo inherited the parental structural rearrangement. Based on CNV and haplotype linkage analyses, we transplanted normal embryos, which were further verified using an amniotic fluid test. Our study is the first to demonstrate the application of the T2T‐CHM13 reference genome to human reproduction. This concept provides a practical scheme for solving the PGT‐SR problems of translocation and inversion, whose breakpoints are located in complex heterochromatin regions, and promotes the medical translation of the T2T‐CHM13 reference genome.

## AUTHOR CONTRIBUTIONS

Zhongyuan Yao, Yanping Li and Sijia Lu conceived and supervised the study. Qiuping Xia and Taoli Ding performed most of the experiments. Taoli Ding, Qiuping Xia and Tianli Chang wrote the manuscript. Jiangxing Ruan, Ji Yang, Jun Ren and Menglin Ma helped draft and review the manuscript. Zhen Liu and Shujing Jiao performed nanopore sequencing. Jiaqi Liu and Jian Wu analysed the data and generated the figures. All the authors have read and approved the final manuscript.

## CONFLICT OF INTEREST STATEMENT

The authors declare no conflict of interest.

## ETHICS STATEMENT

This study was approved by the Medical Ethics Committee of Xiangya Hospital, Central South University (approval number: 2021011). Complete informed consent for all procedures was obtained from all patients. All experiments were performed at the Reproductive Medicine Center of Xiangya Hospital, Central South University, according to previously published procedures. Throughout the study, the collection and use of samples followed procedures that complied with the ethical standards formulated in the Declaration of Helsinki.

## Supporting information

Supporting Information

Supporting Information

Supporting Information

Supporting Information

Supporting Information

Supporting Information

Supporting Information

Supporting Information

Supporting Information

Supporting Information

Supporting Information

Supporting Information

## Data Availability

The data generated or analysed in this study are available in the published article and its Supporting Information files.
