# Supplementary material for: Nanopore sequencing with T2T‐CHM13 for accurate detection and preventing the transmission of structural rearrangements in highly repetitive heterochromatin regions in human embryos
Source: Clin Transl Med. 2024 Mar 6;14(3):e1612. doi: 10.1002/ctm2.1612 (PMC10915734; doi:10.1002/ctm2.1612)
Supplement: Supplementary file 3 — Supporting Information [file CTM2-14-e1612-s004.docx]

**Nanopore sequencing with T2T-CHM13 for accurate detection and blocking of structural rearrangements in highly repetitive heterochromatin regions in human embryos**

Qiuping Xia^1†^, Taoli Ding^2†^, Tianli Chang^1†^, Jiangxing Ruan^2^, Ji Yang^2^, Menglin Ma^2^, Jiaqi Liu^2^, Zhen Liu^2^, Shujing Jiao^2^, Jian Wu^2^, Jun Ren^2^, Sijia Lu^2*^, Yanping Li^1*^ and Zhongyuan Yao^1*^

^1^Reproductive Medicine Center, Xiangya Hospital, Central South University, Changsha, Hunan, 410008, China. ^2^Yikon Genomics Company, Ltd., Suzhou, Jiangsu, 215000, China.

*Corresponding author. Email: [lusijia@yikongenomics.com](mailto:lusijia@yikongenomics.com)[;](mailto:yaozhongyuan@csu.edu.cn;) [lyp7798@126.com](mailto:lyp7798@126.com); [yaozhongyuan@sklmg.edu.cn](mailto:yaozhongyuan@sklmg.edu.cn;)

^†^Qiuping Xia, Taoli Ding, and Tianli Chang contributed equally to this work.

**ACKNOWLEDGEMENTS**

We thank Grandomics and Bena Gene for their assistance with the TGS. We also thank Mr. Jingjing Li, Mr. Shiping Bo, and Dr. Yangyun Zou for their valuable suggestions and data analysis.

**Figure S1.** Integrative Genomics Viewer (IGV) plots of patient 1 displaying the breakpoints of an inversion on chromosome X (chrX). The colored reads indicated support for the inversion, while the gray reads indicated support for the normal haplotype. Additionally, the sequencing depth of each locus was represented by the gray vertical bar. T2T-CHM13, Telomere-to-Telomere (T2T) CHM13.

**Figure S2.** Display of mutation detection results for patient 2. (A) The major filtered mutations that exceed 100 kb. (B) Based on the Telomere-to-Telomere CHM13 (T2T-CHM13) reference genome, the breakpoint position of the translocation on chromosome (chr) 13 was 24,844,058. The colored reads were linked with the pathogenic translocation. (C) The position of the other breakpoint on chromosome 17 was 36,563,186 using the T2T-CHM13 reference genome.

**Supplementary Table 1.** The statistical results of the original detected mutations for patients 1 and 2

**Supplementary Table 2.** The major filtered mutation annotation results for patients 1 and 2

**Supplementary Table 3.** Annotations on structural variations of patient 1 except for translocation

**Supplementary Table 4.** Annotations on translocations of patient 1

**Supplementary Table 5.** Annotations on structural variations of patient 2 except for translocation

**Supplementary Table 6.** Annotations on translocations of patient 2

**Supplementary Table 7.** Phased heterozygous SNPs of patient 1 around the breakpoint of target inversion

**Supplementary Table 8.** Phased heterozygous SNPs of patient 2 & his wife & embryos around the breakpoint of translocation (13q11) using GRCh37

**Supplementary Table 9.** Phased heterozygous SNPs of patient 2 & his wife & embryos around the breakpoint of translocation (17q11.2) using GRCh37
