# Supplementary material for: Nanopore sequencing with T2T‐CHM13 for accurate detection and preventing the transmission of structural rearrangements in highly repetitive heterochromatin regions in human embryos
Source: Clin Transl Med. 2024 Mar 6;14(3):e1612. doi: 10.1002/ctm2.1612 (PMC10915734; doi:10.1002/ctm2.1612)
Supplement: Supplementary file 4 — Supporting Information [file CTM2-14-e1612-s011.docx]

**Supplementary Table 1.** The statistical results of the original detected mutations for patients 1 and 2

| **Mutation type** | **Number of original mutations** | **Number of original mutations** |
| --- | --- | --- |
|  | **patient 1** | **patient 2** |
| Translocation (BND) | 57 | 34 |
| Deletion (DEL) | 14338 | 12807 |
| Duplication (DUP) | 18 | 13 |
| Insertion (INS) | 11678 | 10898 |
| Inversion (INV) | 40 | 38 |
