# Supplementary material for: Nanopore sequencing with T2T‐CHM13 for accurate detection and preventing the transmission of structural rearrangements in highly repetitive heterochromatin regions in human embryos
Source: Clin Transl Med. 2024 Mar 6;14(3):e1612. doi: 10.1002/ctm2.1612 (PMC10915734; doi:10.1002/ctm2.1612)
Supplement: Supplementary file 5 — Supporting Information [file CTM2-14-e1612-s001.docx]

 **Supplementary Table 2.** The major filtered mutation annotation results for patients 1 and 2
