# Supplementary material for: Nanopore sequencing with T2T‐CHM13 for accurate detection and preventing the transmission of structural rearrangements in highly repetitive heterochromatin regions in human embryos
Source: Clin Transl Med. 2024 Mar 6;14(3):e1612. doi: 10.1002/ctm2.1612 (PMC10915734; doi:10.1002/ctm2.1612)
Supplement: Supplementary file 10 — Supporting Information [file CTM2-14-e1612-s009.docx]

**Supplementary Table 7.** Phased heterozygous SNPs of patient 1 around the breakpoint of target inversion

| **SNP** | **Chrom** | **Position** | **patient 1** | |
| --- | --- | --- | --- | --- |
|  |  |  | **hap1** | **hap2** |
| ***rs1135363*** | ***chrX*** | ***100,608,191*** | G | A |
| ***rs3747288*** | ***chrX*** | ***100,611,285*** | T | C |
| ***rs67491067*** | ***chrX*** | ***100,614,597*** | C | T |
| ***rs2746110*** | ***chrX*** | ***100,615,023*** | T | G |
| ***rs5951412*** | ***chrX*** | ***100,615,025*** | T | G |
| ***rs2855259*** | ***chrX*** | ***100,615,478*** | T | C |
| ***rs7053244*** | ***chrX*** | ***100,616,465*** | T | C |
| ***rs5991985*** | ***chrX*** | ***100,616,712*** | A | G |
| ***rs3027641*** | ***chrX*** | ***100,616,989*** | C | T |
| ***rs57578919*** | ***chrX*** | ***100,618,521*** | A | G |
| ***rs112926724*** | ***chrX*** | ***100,618,699*** | A | C |
| ***rs113928932*** | ***chrX*** | ***100,618,702*** | A | C |
| ***rs112012348*** | ***chrX*** | ***100,618,871*** | C | T |
| ***rs3027639*** | ***chrX*** | ***100,620,248*** | G | A |
| ***rs2238987*** | ***chrX*** | ***100,623,072*** | C | T |
| ***rs2301175*** | ***chrX*** | ***100,625,293*** | C | T |
| ***rs6523489*** | ***chrX*** | ***100,627,061*** | A | T |
| ***rs12156856*** | ***chrX*** | ***100,627,901*** | G | A |
| ***rs58911756*** | ***chrX*** | ***100,628,779*** | T | C |
| ***rs112279403*** | ***chrX*** | ***100,628,856*** | C | G |
| ***rs3027623*** | ***chrX*** | ***100,631,138*** | A | G |
| ***rs2238986*** | ***chrX*** | ***100,631,663*** | G | A |
| ***rs2239462*** | ***chrX*** | ***100,632,425*** | T | C |
| ***rs2239461*** | ***chrX*** | ***100,632,637*** | C | T |
| ***rs3027615*** | ***chrX*** | ***100,634,252*** | T | C |
| ***rs7050436*** | ***chrX*** | ***100,634,401*** | G | C |
| ***rs3027607*** | ***chrX*** | ***100,635,063*** | T | C |
| ***rs5951303*** | ***chrX*** | ***100,635,640*** | T | C |
| ***rs112205508*** | ***chrX*** | ***100,636,755*** | T | A |
| ***rs3027613*** | ***chrX*** | ***100,637,128*** | C | T |
| ***rs112741233*** | ***chrX*** | ***100,637,376*** | G | A |
| ***rs3027611*** | ***chrX*** | ***100,637,580*** | G | T |
| ***rs2238984*** | ***chrX*** | ***100,639,801*** | A | G |
| ***rs2071229*** | ***chrX*** | ***100,641,574*** | G | A |
| ***rs5951307*** | ***chrX*** | ***100,644,121*** | G | A |
| ***rs3027600*** | ***chrX*** | ***100,644,567*** | T | C |
| ***rs5951308*** | ***chrX*** | ***100,645,467*** | T | C |
| ***rs113541091*** | ***chrX*** | ***100,648,680*** | C | T |
| ***rs112132208*** | ***chrX*** | ***100,649,408*** | A | T |
| ***rs35019768*** | ***chrX*** | ***100,652,643*** | C | T |
| ***rs2071228*** | ***chrX*** | ***100,653,109*** | G | A |
| ***rs2071397*** | ***chrX*** | ***100,653,950*** | T | C |
| ***rs1023431*** | ***chrX*** | ***100,657,809*** | G | T |
| ***rs111386229*** | ***chrX*** | ***100,659,495*** | C | T |
| ***rs3027586*** | ***chrX*** | ***100,662,219*** | T | C |
| ***rs6621057*** | ***chrX*** | ***100,687,987*** | T | C |
| ***rs6621058*** | ***chrX*** | ***100,687,989*** | T | C |
| ***rs6621059*** | ***chrX*** | ***100,687,999*** | T | C |
| ***rs369421089*** | ***chrX*** | ***100,688,005*** | C | T |
| ***rs182622640*** | ***chrX*** | ***100,709,516*** | C | T |
| ***rs201519615*** | ***chrX*** | ***100,729,476*** | T | C |
| ***rs963618*** | ***chrX*** | ***100,743,037*** | C | T |
| ***rs5951332*** | ***chrX*** | ***100,743,826*** | A | G |
| ***rs5951333*** | ***chrX*** | ***100,745,825*** | T | C |
| ***rs3174476*** | ***chrX*** | ***100,749,585*** | C | T |
| ***rs6523506*** | ***chrX*** | ***100,753,410*** | T | G |
| ***rs7060868*** | ***chrX*** | ***100,753,863*** | T | C |
| ***rs7060883*** | ***chrX*** | ***100,753,922*** | T | C |
| ***rs7060491*** | ***chrX*** | ***100,754,149*** | G | T |
| ***rs2157110*** | ***chrX*** | ***100,754,924*** | C | G |
| ***rs7887626*** | ***chrX*** | ***100,757,661*** | A | G |
| ***rs6621082*** | ***chrX*** | ***100,757,899*** | A | T |
| ***rs376276792*** | ***chrX*** | ***100,758,783*** | T | G |
| ***rs6616250*** | ***chrX*** | ***100,760,103*** | G | A |
| ***rs6621083*** | ***chrX*** | ***100,760,626*** | C | T |
| ***rs5951267*** | ***chrX*** | ***100,767,094*** | T | C |
| ***rs5951339*** | ***chrX*** | ***100,767,126*** | C | T |
| ***rs55917049*** | ***chrX*** | ***100,767,185*** | A | G |
| ***rs1114843*** | ***chrX*** | ***100,767,857*** | G | A |
| ***rs2361299*** | ***chrX*** | ***100,767,860*** | G | T |
| ***rs36017889*** | ***chrX*** | ***100,768,592*** | G | A |
| ***rs67235608*** | ***chrX*** | ***100,769,910*** | T | A |
| ***rs72615580*** | ***chrX*** | ***100,770,514*** | C | A |
| ***rs5951340*** | ***chrX*** | ***100,771,055*** | T | C |
| ***rs5951268*** | ***chrX*** | ***100,772,491*** | A | G |
| ***rs5951341*** | ***chrX*** | ***100,773,518*** | C | A |
| ***rs5951342*** | ***chrX*** | ***100,773,590*** | A | G |
| ***rs5951343*** | ***chrX*** | ***100,774,559*** | T | C |
| ***rs190032386*** | ***chrX*** | ***100,776,499*** | T | G |
| ***rs5951269*** | ***chrX*** | ***100,778,274*** | C | T |
| ***rs2057354*** | ***chrX*** | ***100,778,863*** | G | T |
| ***rs2057355*** | ***chrX*** | ***100,779,320*** | A | G |
| ***rs6523509*** | ***chrX*** | ***100,779,655*** | C | A |
| ***rs5951344*** | ***chrX*** | ***100,779,664*** | C | T |
| ***rs141163587*** | ***chrX*** | ***100,781,515*** | C | T |
| ***rs148942171*** | ***chrX*** | ***100,782,270*** | A | G |
| ***rs148078529*** | ***chrX*** | ***100,783,746*** | A | G |
| ***rs188350543*** | ***chrX*** | ***100,784,211*** | C | A |
| ***rs12833777*** | ***chrX*** | ***100,785,073*** | A | T |
| ***rs5951349*** | ***chrX*** | ***100,785,918*** | A | G |
| ***rs5991944*** | ***chrX*** | ***100,792,633*** | C | T |
| ***rs7878970*** | ***chrX*** | ***100,793,168*** | A | G |
| ***rs6523510*** | ***chrX*** | ***100,793,995*** | G | C |
| ***rs1967744*** | ***chrX*** | ***100,794,731*** | C | T |
| ***rs200187824*** | ***chrX*** | ***100,798,582*** | A | T |
| ***rs139047275*** | ***chrX*** | ***100,798,650*** | T | A |
| ***rs200504230*** | ***chrX*** | ***100,799,265*** | T | G |
| ***rs374228263*** | ***chrX*** | ***100,799,601*** | A | G |
| ***rs141807963*** | ***chrX*** | ***100,799,920*** | C | G |
| ***rs141933626*** | ***chrX*** | ***100,799,921*** | A | G |
