# Supplementary material for: Nanopore sequencing with T2T‐CHM13 for accurate detection and preventing the transmission of structural rearrangements in highly repetitive heterochromatin regions in human embryos
Source: Clin Transl Med. 2024 Mar 6;14(3):e1612. doi: 10.1002/ctm2.1612 (PMC10915734; doi:10.1002/ctm2.1612)
Supplement: Supplementary file 11 — Supporting Information [file CTM2-14-e1612-s002.docx]

**Supplementary Table 8.** Phased heterozygous SNPs of patient 2 & his wife & embryos around the breakpoint of translocation (13q11) using GRCh37

| **ID** | **Chromosome** | **Distance** | **patient 2's wife** | | **patient 2** | | **A** | | **B** | | **C** | | **K** | | **L** | |
| --- | --- | --- | --- | --- | --- | --- | --- | --- | --- | --- | --- | --- | --- | --- | --- | --- |
|  |  |  | **Hap1** | **Hap2** | **Hap1** | **Hap2** | **Hap1** | **Hap2** | **Hap1** | **Hap2** | **Hap1** | **Hap2** | **Hap1** | **Hap2** | **Hap1** | **Hap2** |
| rs12429015 | chr13 | -1,629,450 | G | G | A | G | A | G | G | G | G | G | G | G | A | G |
| rs9553164 | chr13 | -1,546,460 | T | T | T | G | T | T | 0 | 0 | G | T | 0 | 0 | T | T |
| rs9511088 | chr13 | -1,488,900 | C | C | C | T | C | C | T | C | T | C | T | C | C | C |
| rs7333431 | chr13 | -1,488,200 | A | A | A | G | A | A | G | A | G | A | G | A | A | A |
| rs2031162 | chr13 | -1,474,880 | T | T | T | C | T | T | C | T | C | T | C | T | T | T |
| rs7318203 | chr13 | -1,411,930 | C | C | T | C | T | C | C | C | C | C | C | C | T | C |
| rs74860689 | chr13 | -1,305,790 | G | G | A | G | 0 | 0 | G | G | G | G | G | G | 0 | 0 |
| rs78280928 | chr13 | -1,298,400 | G | G | A | G | A | G | G | G | 0 | 0 | G | G | A | G |
| rs9507310 | chr13 | -1,292,750 | A | A | G | A | G | A | A | A | A | A | A | A | G | A |
| rs1050112 | chr13 | -1,199,000 | G | G | T | G | T | G | G | G | G | G | G | G | T | G |
| rs13428 | chr13 | -1,198,860 | C | C | G | C | G | C | C | C | C | C | C | C | 0 | 0 |
| rs943055 | chr13 | -1,006,390 | C | C | A | C | A | C | C | C | C | C | C | C | A | C |
| rs9511698 | chr13 | -423,470 | G | G | A | G | A | G | G | G | G | G | G | G | A | G |
| rs9553561 | chr13 | -416,120 | G | G | A | G | A | G | G | G | G | G | G | G | A | G |
| rs4769420 | chr13 | -220,230 | C | C | T | C | T | C | C | C | C | C | C | C | T | C |
| rs17686146 | chr13 | 106,860 | T | T | T | C | T | T | C | T | C | T | C | T | T | T |
| rs2181426 | chr13 | 159,050 | A | A | A | G | A | A | G | A | G | A | G | A | A | A |
| rs3783124 | chr13 | 249,940 | T | T | T | C | T | T | C | T | C | T | C | T | T | T |
| rs306416 | chr13 | 285,180 | G | G | G | A | G | G | A | G | A | G | A | G | G | G |
| rs9551232 | chr13 | 392,640 | G | G | A | G | A | G | G | G | G | G | G | G | A | G |
| rs9553706 | chr13 | 399,370 | T | T | C | T | C | T | T | T | T | T | T | T | * | * |
| rs12430901 | chr13 | 469,440 | T | T | T | C | T | T | C | T | C | T | C | T | T | T |
| rs139760119 | chr13 | 476,970 | A | A | G | A | G | A | A | A | A | A | A | A | 0 | 0 |
| rs9507713 | chr13 | 808,140 | A | A | A | G | A | A | G | A | G | A | G | A | A | A |
| rs979244 | chr13 | 881,740 | C | C | C | T | C | C | T | C | T | C | T | C | C | C |
| rs9512257 | chr13 | 906,030 | A | A | A | C | A | A | C | A | C | A | C | A | A | A |
| rs138093490 | chr13 | 909,320 | A | A | G | A | G | A | A | A | A | A | A | A | G | A |
| rs60934898 | chr13 | 975,890 | G | G | G | A | G | G | A | G | A | G | A | G | G | G |
| rs74531891 | chr13 | 1,097,580 | C | C | T | C | T | C | C | C | C | C | C | C | T | C |
| rs11618271 | chr13 | 1,228,660 | C | C | T | C | 0 | 0 | C | C | C | C | C | C | 0 | 0 |
