# Supplementary material for: Nanopore sequencing with T2T‐CHM13 for accurate detection and preventing the transmission of structural rearrangements in highly repetitive heterochromatin regions in human embryos
Source: Clin Transl Med. 2024 Mar 6;14(3):e1612. doi: 10.1002/ctm2.1612 (PMC10915734; doi:10.1002/ctm2.1612)
Supplement: Supplementary file 12 — Supporting Information [file CTM2-14-e1612-s010.docx]

**Supplementary Table 9.** Phased heterozygous SNPs of patient 2 & his wife & embryos around the breakpoint of translocation (17q11.2) using GRCh37

| **ID** | **Chromosome** | **Distance** | **patient 2's wife** | | **patient 2** | | **A** | | **B** | | **C** | | **K** | | **L** | |
| --- | --- | --- | --- | --- | --- | --- | --- | --- | --- | --- | --- | --- | --- | --- | --- | --- |
|  |  |  | **Hap1** | **Hap2** | **Hap1** | **Hap2** | **Hap1** | **Hap2** | **Hap1** | **Hap2** | **Hap1** | **Hap2** | **Hap1** | **Hap2** | **Hap1** | **Hap2** |
| rs1437214 | chr17 | -1,113,980 | A | A | G | A | A | A | G | A | G | A | 0 | 0 | A | A |
| rs4795931 | chr17 | -1,101,060 | G | G | G | A | A | G | 0 | 0 | G | G | G | G | A | G |
| rs17662217 | chr17 | -1,080,090 | T | T | T | C | C | T | T | T | T | T | T | T | C | T |
| rs73988732 | chr17 | -1,012,640 | A | A | C | A | A | A | C | A | C | A | C | A | A | A |
| rs16970090 | chr17 | -1,005,740 | G | G | A | G | G | G | A | G | A | G | A | G | G | G |
| rs7208546 | chr17 | -973,870 | G | G | A | G | G | G | A | G | A | G | A | G | G | G |
| rs62062166 | chr17 | -972,420 | G | G | A | G | G | G | A | G | A | G | A | G | G | G |
| rs1860277 | chr17 | -970,280 | T | T | T | C | C | T | T | T | T | T | T | T | C | T |
| rs4795955 | chr17 | -968,210 | A | A | G | A | A | A | G | A | G | A | G | A | A | A |
| rs8182290 | chr17 | -963,260 | G | G | A | G | G | G | A | G | A | G | A | G | G | G |
| rs117524440 | chr17 | -786,550 | T | T | T | C | C | T | T | T | T | T | T | T | * | * |
| rs2074517 | chr17 | -617,600 | T | T | T | C | C | T | T | T | T | T | T | T | C | T |
| rs3744365 | chr17 | -428,690 | A | A | A | C | C | A | A | A | A | A | A | A | C | A |
| rs76268785 | chr17 | -329,910 | T | T | C | T | T | T | C | T | C | T | C | T | T | T |
| rs8078071 | chr17 | -242,510 | C | C | T | C | C | C | T | C | T | C | T | C | 0 | 0 |
| rs373440543 | chr17 | 243,040 | C | C | C | T | T | C | C | C | C | C | C | C | T | C |
| rs11651172 | chr17 | 328,010 | G | G | G | A | A | G | G | G | G | G | G | G | A | G |
| rs760191 | chr17 | 328,270 | C | C | C | A | 0 | 0 | C | C | C | C | C | C | 0 | 0 |
| rs79254649 | chr17 | 362,320 | C | C | A | C | C | C | A | C | A | C | 0 | 0 | C | C |
| rs74842203 | chr17 | 362,400 | C | C | T | C | C | C | T | C | T | C | 0 | 0 | 0 | 0 |
| rs854684 | chr17 | 369,930 | T | T | T | C | C | T | T | T | T | T | T | T | C | T |
| rs17617750 | chr17 | 503,260 | C | C | C | T | T | C | C | C | C | C | C | C | T | C |
| rs757121 | chr17 | 513,100 | C | C | C | T | T | C | C | C | C | C | C | C | T | C |
| rs12949233 | chr17 | 1,114,100 | A | A | A | G | G | A | A | A | A | A | A | A | G | A |
| rs7224858 | chr17 | 1,123,040 | G | G | A | G | G | G | A | G | A | G | A | G | G | G |
| rs17138064 | chr17 | 1,246,810 | G | G | G | A | A | G | G | G | G | G | G | G | A | G |
| rs79559078 | chr17 | 1,342,450 | C | C | C | A | A | C | C | C | C | C | C | C | 0 | 0 |
| rs76472876 | chr17 | 2,638,190 | C | C | C | T | T | C | C | C | C | C | C | C | * | * |
| rs2502364 | chr17 | 2,757,040 | A | A | A | G | G | A | A | A | A | A | A | A | * | * |
| rs1984723 | chr17 | 2,963,200 | G | G | A | G | G | G | 0 | 0 | A | G | 0 | 0 | G | G |
